# Relationships between gross motor skills, psychological resilience, executive function, and emotional regulation among Chinese rural preschoolers: A moderated mediation model

**DOI:** 10.1016/j.heliyon.2024.e38039

**Published:** 2024-09-19

**Authors:** Xinyue Ma, Ning Yang, Meixian Huang, Shuwei Zhan, Houwen Cao, Shan Jiang

**Affiliations:** a Department of Early Childhood Education, Guangdong Teachers College of Foreign Languages and Arts, Guangzhou, Guangdong Province, China; b Department of Education, South China Normal University, Guangzhou, Guangdong Province, China; c Department of Education, Central China Normal University, Wuhan, Hubei Province, China; d School of Kinesiology and Health Promotion, Dalian University of Technology, Dalian, China; e Department of Sports Science and Physical Education, The Chinese University of Hong Kong, Shatin, Hong Kong

**Keywords:** Preschooler, Gross motor skills, Emotional regulation, Executive function, Psychological resilience

## Abstract

**Background:**

Emotional regulation is a critical component of emotional intelligence, particularly during the preschool stage, a key period for children's development. Previous studies have demonstrated that executive function mediates the effect of gross motor skills on emotional understanding. However, studies specifically focusing on children from rural areas and investigating the role of psychological resilience are limited. The present study fills this knowledge gap by examining the effect of gross motor skills on emotional regulation and the roles of executive function and psychological resilience among Chinese rural preschool children.

**Methods:**

This study included 430 children (aged 61.01 ± 6.98 months, 48.8 % boys) and their teachers from three rural preschools in China. Children's gross motor skills, including locomotor and object control skills, were assessed using the Test of Gross Motor Development-3. Executive function was measured using the Head-Toes-Knee-Shoulder task, and emotional regulation was assessed using the Emotional Regulation Checklist. Furthermore, psychological resilience was examined using the Devereux Early Childhood Assessment. Demographic information was collected, and the cross-sectional relationships between gross motor skills and emotional regulation were investigated through mediation and moderation analyses.

**Results:**

Gross motor skills, executive function, and psychological resilience were associated with emotional regulation (p < 0.05), after controlling for sex, age, and only-child status. Executive function was found to mediate the relationship between gross motor skills and emotional regulation, with a mediation effect of 0.045. Psychological resilience moderated the relationship between executive function and emotional regulation (β = 0.078, p < 0.05). Simple slope analysis, based on categorizing psychological resilience into high, medium, and low groups, revealed that preschoolers with a higher level of psychological resilience exhibited a significantly stronger predictive effect of executive function on emotional regulation (β = 0.202, p < 0.01).

**Conclusions:**

Gross motor skills significantly affect emotional regulation development in rural preschoolers, with executive function acting as a mediator in this relationship. Psychological resilience was found to moderate the effect of executive function on emotional regulation. The findings suggest that enhancing gross motor skills through physical activities can benefit children by promoting the development of executive function, which is crucial for emotional regulation. On the basis of our findings, we recommend focusing on cost-effective physical activity interventions for motor skills development among rural children while also addressing the development of executive function and psychological resilience. Future efforts should include workshops to improve physical literacy of parents and teachers regarding children's gross motor skills promotion.

## 1 Introduction

Emotional regulation refers to the process through which individuals manage, monitor, assess, and adjust their emotions. This process encompasses both internalization and externalization, including emotional arousal, feedback, and control [1,2]. The ability to regulate emotions is a crucial aspect of emotional intelligence development, with the preschool stage being a pivotal period for developing these skills [3]. Children who receive better care and more stimulation from caregivers at home typically exhibit substantial improvements in their emotional and social skill development [4]. However, studies have consistently reported that the home environment in rural China often lacks these benefits, and marked disparities exist between rural and urban areas [5,6]. These disparities include higher socioeconomic risks and less supportive emotional contexts within families [7,8]. For instance, Zeng et al. (2009) examined 3,944 left-behind children aged 4–7 years in rural China and determined that 43.6 % of these children exhibited behavioral and emotional problems [9]. Children with poor emotional regulation abilities may encounter challenges in peer relationships, experience lower levels of peer acceptance, and have a higher likelihood of displaying internalizing (e.g., social withdrawal, anxiety, and depression) and externalizing behaviors (e.g., aggressive conduct) [10]. In summary, although existing studies have identified factors affecting emotional regulation, studies specifically focusing on rural preschool children and examining the complex interactions among different influencing factors are scant. Thus, the present study identified key factors and mechanisms affecting emotional regulation in rural preschool children. The findings of this study can facilitate the development of effective support strategies to enhance the emotional regulation ability of these children.

According to the Social-Emotional Selection, Optimization, and Compensation Theory, various factors affect the selection and use of emotional regulation strategies [11]. Among these factors, motor skills are identified as a critical protective element in the development of emotional abilities. This is particularly evident in research on special populations. For example, Battaglia investigated the efficacy of a multisystemic aquatic therapy in enhancing gross motor skills and social behaviors in adolescents with autism spectrum disorders [12]. However, studies examining the role of motor skills in emotional regulation among rural preschool children and the effects of other factors, such as social background [[13], [14]] and cultural influences [15], on the relationship between motor skills and emotional regulation are limited. Considering these potential influencing factors, especially those related to psychological development, is crucial. The present study investigated internal mechanisms through which motor skills affect emotional regulation development in rural preschool children and examined whether these effects are mediated or moderated by executive function and psychological resilience.

### 1.1 Gross motor skills and emotional regulation

Gross motor skills refer to the fundamental patterns of non-naturally occurring body movements that lay the groundwork for participating in complex physical and sports activities. These skills include locomotor skills (e.g., running and jumping), object control skills (e.g., hitting a ball and catching), and stability skills [16]. The preschool years are crucial for children to acquire these fundamental movement skills, which are indispensable to the development of broader motor skills [[17], [18]]. In addition, these skills are positively associated with various aspects of health development, including physical activity [19], motor coordination [20], food literacy [21], and social and cognitive abilities [22,23]. The development of children's emotional regulation also progresses alongside their motor skills. Neurophysiological studies have indicated that enhancing motor skills is an economical and effective method for managing anxiety and improving emotional regulation [24]. Motor activities that involve diverse movement skills activate the prefrontal cortex of the brain, promoting the release of neurotransmitters such as dopamine, norepinephrine, serotonin, endorphins, and opioids, thereby improving mood. In addition to theoretical analyses, empirical studies examining the relationship between motor skills and emotional regulation should be conducted. Thompson reviewed studies on emotional regulation development and found that the maturation of an infant's nervous system aligns with the progression from basic self-soothing behaviors, such as finger-sucking, to more advanced regulatory methods, such as controlling visual attention and behavioral avoidance. This progression is closely linked to children's motor skills [25]. Reed and Buck reviewed 105 studies investigating the effect of physical activity interventions on emotions and found that participants in the experimental group were more likely to experience positive emotions compared with those in the control group [26]. Physical activities increase dopamine levels, positively affecting mood regulation and behavior. On the basis of these findings, the present study proposes hypothesis H1: Gross motor skills significantly and positively predict emotional regulation.

#### 1.1.1 Mediating role of executive function

Executive function involves a range of higher-order cognitive processes essential for regulating and managing goal-directed behavior. Executive function includes working memory, inhibitory control, and cognitive flexibility, all of which are associated with the functioning of the prefrontal cortex [[27], [28]]. Executive function is crucial for children's mental health, learning abilities, and academic performance [29]. Motor skills substantially affect executive function, and enhancing motor skills is a practical and effective method for promoting the development of executive function [30,31].

From a cognitive neuroscience perspective, motor skills and executive function share neural mechanisms in various brain regions, such as the prefrontal cortex and cerebellum [32]. Empirical evidence indicates that children with higher levels of motor skills tend to have better developed executive function [33,34]. From a cognitive psychology perspective, although motor skills are not traditionally viewed as part of the cognitive domain, Piaget argued that the term “action” involves cognitive elements, especially in the early childhood perceptual-motor stage, where the motor and cognitive skills share several common underlying processes, including organization, joint observation, and planning [35].

Emotional regulation and executive function are closely associated. The interactive model proposed by Zelazo and colleagues suggests that emotional regulation and executive function are closely linked, either occurring simultaneously or sequentially, and are deeply integrated [36]. Empirical studies have indicated that children with well-developed executive function are more likely to use positive emotional regulation strategies [37]. By contrast, deficits or dysregulation in executive function may be associated with emotional problems, such as depression, anxiety, sensitivity, and irritability [38,39].

Although motor skills can affect both executive function and emotional regulation, a key question is whether executive function mediates the relationship between fundamental motor skills and emotional regulation. Neuroscientific research indicates that brain mechanisms underlying motor skills, emotional regulation, and executive function partially overlap in regions such as the anterior cingulate cortex, ventrolateral prefrontal cortex, and dorsolateral prefrontal cortex. These shared neural mechanisms affect information processing and responses [40,41]. However, empirical research confirming the mediation role of executive function in this relationship is lacking. On the basis of these findings, the second research hypothesis H2 is proposed: Executive function mediates the relationship between gross motor skills and emotional regulation.

#### 1.1.2 Regulatory role of psychological resilience

Not all children facing adverse situations follow a linear model of “adverse circumstances (high risk) - stress - maladaptive adaptation.” However, individuals with a high level of psychological resilience can effectively mitigate the negative effects of these challenging circumstances [41,42]. Psychological resilience refers to an individual's ability to adapt to and manage their self-states in response to internal conflicts and psychological needs, demonstrating flexibility and effective coping strategies [43].

Psychological resilience is affected by various factors and undergoes a dynamic developmental process, with emotional regulation playing a central role [44]. Zhang found that children with a high level of psychological resilience are adept at providing rational emotional responses to challenging situations and using positive emotional regulation strategies [45]. Furthermore, psychological resilience is closely related to executive function [46]. The self-efficacy-based model of psychological resilience posits that self-efficacy is a critical component of psychological resilience [47]. Given that executive function affects the goal-directed behavior, it is highly positively correlated with self-efficacy [48]. Obradović found that executive function skills can enhance psychological resilience development in children from disadvantaged socioeconomic backgrounds, including homeless children [49].

The Protective-Protective Model theory proposed that different protective factors can interact when predicting developmental outcomes. According to this theory, the effect of one protective factor (e.g., executive function) on an outcome variable (e.g., emotional regulation) may be moderated by another protective factor (e.g., psychological resilience). On the basis of this concept, we propose the third hypothesis H3: Psychological resilience moderates the relationship between executive function and emotional regulation.

##### 1.1.2.1 Study context

Developmental delays are prevalent among young children in developing countries [50]. Nearly 249.4 million children under the age of 5 years have a risk of not reaching their developmental potential [51], and over 80 million children aged 3–4 years have a risk of poor cognitive and socioemotional development due to poverty, inadequate health and nutrition, and insufficient caregiving [52]. In Western countries, interventions such as mindful parenting training have demonstrated effectiveness in enhancing emotional regulation [53,54]. However, in rural China, implementing parent intervention programs for children's emotional regulation is challenging because of limited resources, lower socioeconomic status, and impoverished family environments, which make children more vulnerable to difficulties in emotional regulation [55,56]. Given these challenges, factors and mechanisms affecting emotional regulation in school settings in rural China should be explored. Understanding how emotional regulation, executive function, psychological resilience, and gross motor skills may differ between Chinese and Western settings can provide valuable insights and highlight the need for empirical research in the rural Chinese context to thoroughly investigate these dynamics [57,58].

**Fig. 1:**
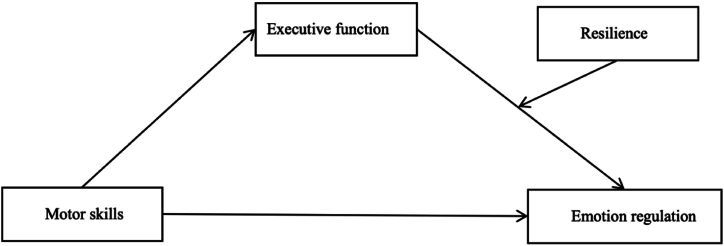
Hypothesized moderated mediation model.

After reviewing the literature, we propose a moderated mediation model to elucidate the specific mechanisms involved (Fig. 1). The hypotheses are as follows.

H1 Motor skills significantly predict emotional regulation.

H2 Executive function mediates the relationship between motor skills and emotional regulation.

H3 Psychological resilience moderates the mediating effect of executive function on the relationship between motor skills and emotional regulation.

## 2 Materials and methods

### 2.1 Study design and participants

Participants were randomly selected from Guangdong Province, a densely populated area in Southern China with a significant migrant worker population and rapid economic development. The study focused on economically underdeveloped and rural areas within the province, targeting regions with a high concentration of preschool children. Using data from the provincial economic geography database, six kindergartens in three impoverished areas were identified. This study was conducted between March and May 2022 and focused on rural Chinese preschool children aged 4–6 years, reflecting the region's economic challenges, low parental educational awareness, and typical kindergarten entry age. The testing was conducted within the same period to avoid the influence of seasonal and temporal factors on the results. Parents were informed in advance and asked to dress their children in sportswear on the test day to ensure their safety during the tests, and they provided basic information about their children beforehand. The DECA-P2 tool, designed to assess resilience in children aged 3–5 years, was used to evaluate the participants. Children (a) aged 4–6 years, (b) having resided in rural areas for at least 6 months, and (c) with good physical and mental health were recruited. Children whose caregivers had communication impairments, such as cognitive problems or deafness, and those with severe physical or mental illnesses were excluded.

The study involved 440 children and 72 female teachers from the six selected kindergartens. The sample size for this study was determined using GPower (version 3.1.9.2), developed by Franz Faul at the University of Kiel, Germany [59]. Based on an alpha level of 0.05 and a power level of 0.80, a minimum of 352 participants was deemed necessary for the models. This sample size was considered sufficient to meet the study's requirements. In practice, data were collected from 450 participants across all kindergartens, with a total of 75 preschoolers per kindergarten. However, after careful examination, 430 valid samples were obtained, resulting in an effective sample rate of 95.6 %. The validity of the samples was ensured by adhering to several criteria [1]: missing data did not exceed three items [2], respondents did not consistently select the same option for all items, and data for all four variables were complete [3].

### 2.2 Procedures

Prior to commencing the project, ethical approval was obtained from the Ethics Committee of Anhui Normal University (AHNU-ET2021031). A professionally trained research team was assembled and conducted a standardized recruitment seminar. This seminar outlined the research objectives, procedures, and instructions for completing the questionnaire to the participants, including kindergarten principals, teachers, and parents. After the seminar, all participants, including parents who informed their children and obtained their consent, voluntarily signed informed consent forms and provided their contact information for further communication.

The study employed various tests and questionnaires to collect data. Preschool teachers completed questionnaires on resilience and emotional regulation for each child, whereas researchers administered tests for motor skills and executive function. All instruments and questionnaires were in Chinese and validated for research in China by early childhood education professionals. Parents were instructed to dress their children in sportswear for safety during the tests and to provide basic information about their children. For example, during the Test of Gross Motor Development-3 (TGMD-3) test, children were encouraged to participate actively. Trained demonstrators showed each movement twice, and the children then imitated and performed the movements twice. These sessions were videotaped for evaluation. Performance was assessed using qualitative criteria, with scores of 1 indicating the presence of a criterion and 0 indicating its absence. The raw scores ranged from 0 to 6 based on the number of criteria met. Four trained researchers (ZPC, YYY, HMX, and ZQY) scored the TGMD-3 assessments, achieving an agreement rate of over 90 %. To minimize bias, all data assessors, investigators, TGMD-3 raters, parents, and children were blinded to the primary outcomes and hypotheses of the study.

## 3 Measurements

### 3.1 Gross motor skills

The TGMD-3 is the most widely used assessment tool for evaluating motor skills in children aged 3–10 years [60]. This scale assesses both locomotor skills and object control skills [61]. Locomotor skills include running, galloping, sliding, skipping, horizontal jumping, and hopping. Object control skills include two-hand catching, kicking a stationary ball, one-hand stationary dribbling, forehand striking of a self-bounced ball, overhand throwing, underhand throwing, and two-hand striking of a stationary ball. The TGMD-3 assesses a total of 13 movements, each with 3–5 scoring criteria. Each movement is performed twice, and the scores are cumulative. The maximum score is 46 for the six locomotor skills and 54 for the seven object control skills. The total maximum score is 100 [62]. In this study, Cronbach's α coefficients for locomotor skills and object control skills were 0.862 and 0.729, respectively, indicating good internal consistency for each dimension.

### 3.2 Emotional regulation

The Emotion Regulation Checklist (ERC) is a widely used tool for assessing emotion regulation in preschool children and has been employed globally [63,64]. The ERC consists of two scales: the Emotion Regulation Scale and the Lability/Negativity Scale. The Emotion Regulation Scale measures appropriate emotional expressions, empathy, and emotional self-awareness (e.g., “Shows understanding of others' feelings”) and includes 10 items. The Lability/Negativity Scale assesses the negative effects of emotional instability, inflexibility, and dysregulation (e.g., “Exhibits wide mood swings” and “Easily angered”) and includes 14 items. Teachers rate each item on a 4-point Likert scale (1 = never, 2 = sometimes, 3 = often, 4 = almost always). For statistical analysis, the Lability/Negativity Scale was reverse scored to create the Emotion Stability Scale. The total emotion regulation score was obtained by combining the scores from both scales, with a higher total score indicating greater effectiveness in emotional regulation. In this study, Cronbach's α coefficients were 0.778 for emotional regulation and 0.713 for emotional stability, indicating acceptable internal consistency for each dimension.

### 3.3 Executive function

The Head-Toes-Knee-Shoulder (HTKS) task is used to assess executive function in children [65] and consists of three levels of difficulty [1]: Head-Toes Task (HTT) [2], Head-Toes-Knee-Shoulder Task (HTKS), and [3] Head-Toes-Knee-Shoulder Task with additional rule changes (HTKS-Extension). The HTKS task includes six practice tests and 30 test items that assess various aspects of executive function, such as attention to instructions, working memory to remember and execute new rules, inhibitory control to suppress dominant responses, and cognitive flexibility to adapt to new rules in subsequent rounds. Children must score ≥4 points in a section to proceed to the next level, with the total score ranging from 0 to 60. A higher score indicates a higher level of executive function.

### 3.4 Psychological resilience

The Devereux Early Childhood Assessment (DECA) is a tool used to evaluate children's resilience across three dimensions: initiative, self-control, and attachment/relationship [[66], [67]]. The initiative dimension includes 9 items (e.g., “persists in tasks even when difficult”). The self-control dimension also includes 9 items (e.g., “controls anger”). The attachment/relationship dimension consists of 9 items (e.g., “Child's behavior makes adults laugh or gets their attention”). In addition, the behavioral concern dimension includes 11 items (e.g., “becomes easily distracted”). Each item is rated on a 5-point Likert scale (0 = never, 1 = rarely, 2 = occasionally, 3 = frequently, 4 = consistently). The children's main classroom teacher completes the assessment, and higher scores on the protective factors indicate greater psychological resilience. In this study, Cronbach's α coefficients for initiative, self-control, and attachment/relationship were 0.842, 0.844, and 0.768, respectively, indicating good internal consistency for each dimension.

### 3.5 Statistical analyses

Data were analyzed using SPSS 26.0 (IBM Corp., Armonk, NY, USA, 2021) [68]. First, descriptive statistics and correlation analyses were conducted to examine the relationships between variables. Next, the Hayes PROCESS model was used to test the moderated mediation model [69]. To obtain bias-corrected 95 % confidence intervals, the bootstrap method was applied with 5,000 resamples. In addition, an exploratory factor analysis was performed on the teacher-rated questionnaires for resilience and emotional regulation. Harman's single-factor test identified 14 factors with eigenvalues greater than 1, with the maximum variance explained by any single factor being 20.38 %, well below the 40 % threshold. Thus, the study did not exhibit significant common method bias [70].

## 4 Results

### 4.1 Descriptive statistics and bivariate correlation analysis

Table 1 lists the demographic information of the participants. Table 2 presents the descriptive statistics, including the mean, standard deviation, and correlation matrix for each variable. The findings revealed significant positive correlations between gross motor skills and both executive function and emotional regulation. Furthermore, emotional regulation was positively correlated with executive function, psychological resilience, and motor skills. Among demographic variables, gender was significantly positively correlated with executive function, and age was positively correlated with both executive function and motor skills. Furthermore, a child's status as “left behind” was significantly positively correlated with emotional regulation and motor skills. Based on these findings, gender, age, and left-behind status were included as control variables in subsequent analyses.

**Table 1:** Participants' demographic information.

Variables		Frequency	Percentage	Effective percentage	Cumulative percentage
Gender	Male	210	48.8	48.8	48.8
	Female	220	51.2	51.2	100
only child status	Yes	192	44.7	44.7	44.7
	No	238	55.3	55.3	100
Age	4 years old	207	48.1	48.1	48.1
	5 years old	223	51.9	51.9	100
Total		430

**Table 2:** Mean, standard deviation and correlation coefficients of variables (N = 430).

Variables	1	2	3	4	5	6	M	SD
1.Gender								
2.Age	0.039							
3.Only child status	0.028	0.04						
4.motor skills	−0.03	0.326∗∗	−0.227∗∗				44.684	10.24
5.Exective function	0.105∗	0.382∗	−0.074	0.300∗∗			21.295	17.579
6.Resilience	0.043	−0.028	−0.023	0.089	0.160∗∗		87.558	13.509
7.Emotion regulation	0.074	−0.079	0.455∗∗	0.204∗∗	0.241∗∗	0.160∗∗	72.99	6.798

Note: ∗∗∗p < 0.001,∗∗p < 0.01,∗p < 0.05.

### 4.2 Mediation analysis

To determine the relationships between variables, continuous variables were standardized (see Table 3). We first employed the Hayes PROCESS Model 4 to test the mediation effect, with gender, left-behind status, and age included as control variables. A bootstrap method with 5,000 resamples was used to estimate the 95 % confidence interval for the mediation effect. This analysis was performed to determine the effect size and confidence interval for the mediation effect of executive function between gross motor skills and emotional regulation.

**Table 3:** Test of the mediating effect of resilience.

Variables	Model 1：Emotion regulation				Model 2：Exective function				Model 3：Emotion regulation			
	β	t	Bootstrap95%CI		β	t	Bootstrap95%CI		β	t	Bootstrap95%CI	
Gender	−0.164	−2.302	−0.272	0.082	−0.152	−1.811	−0.317	0.013	−0.164	−1.955	−0.406	−0.041
Only child status	0.085	0.963	−0.088	0.258	0.045	0.516	−0.126	0.215	0.075	0.873	−0.094	0.245
Age	−0.095	−1.059∗∗∗	−0.272	0.082	0.601	6.78∗∗∗	0.426	0.775	−0.224	−2.412	−0.406	−0.041
motor skills	0.22	4.152∗∗∗	0.047	0.248	0.212	4.068∗∗∗	0.109	0.314	0.174	3.304∗∗∗	0.07	0.278
Emotion regulation									0.214	4.428∗∗∗	0.119	0.309
R2	0.059				0.188				0.1			
F	6.640∗∗∗				24.575∗∗∗				9.464∗∗∗			

Note: ∗∗∗p < 0.001,∗∗p < 0.01,∗p < 0.05.

The mediation effect testing results (Table 4) indicated that gross motor skills significantly predicted emotional regulation (β = 0.22, t = 4.152, p < 0.01). When executive function was included as a mediator, the direct predictive effect of gross motor skills on emotional regulation remained significant (β = 0.174, t = 3.304, p < 0.01). In addition, as shown in Table 5, the 95 % confidence interval for the mediation effect did not include 0 (BootLL CI = 0.022, BootULCI = 0.073), indicating that executive function serves as a mediator between gross motor skills and emotional regulation, with a mediation effect of 0.045.

**Table 4:** Decomposition table of the total effect, direct effect, and mediating effect.

Types of Effect	Effect	BootSE	Bootstrap95%CI		Relative effect value
			BootLLCI	BootULCI	
Total effect	0.22	0.053	0.116	0.324	
Direct effect	0.174	0.053	0.071	0.278	79.09 %
mediating effect	0.045	0.013	0.022	0.073	20.45 %

**Table 5:** Test of the moderated mediation effects of motor skills on emotion regulation.

Variables	Model 1：Exective function				Model 2：Emotion regulation			
	β	t	Bootstrap95%CI		β	t	Bootstrap95%CI	
			BootLLCI	BootULCI			BootLLCI	BootULCI
Gender	−0.152	−1.811	−0.317	0.013	−0.137	−1.959	−0.274	0
Only child status	0.045	0.516	−0.126	0.215	0.067	0.932	−0.074	0.209
Age	0.6	6.782∗∗∗	0.427	0.775	−0.102	−1.312	−0.255	0.051
motor skills	0.216	4.068∗∗∗	0.109	0.314	0.126	2.863∗	0.04	0.213
Exective function					0.124	3.040∗	0.044	0.204
Resilience					0.493	13.61∗∗∗	0.422	0.565
Exective function × Resilience					0.078	2.164∗	0.007	0.149
R2	0.188				0.379			
F	24.575∗∗∗				36.825∗∗∗			

After controlling for the mediating variable (executive function), we determined that gross motor skills continued to significantly affect emotional regulation, with the confidence interval for the direct effect not including 0 (BootLLCI = 0.071, BOOTULCI = 0.278). The direct effect of 0.174 and the mediation effect of 0.045 accounted for 79.09 % and 20.45 %, respectively, of the total effect (0.22). These findings indicate that executive function mediates the relationship between gross motor skills and emotional regulation, confirming Hypothesis 2.

### 4.3 Moderation analyses

Continuing the analysis with the SPSS PROCESS macro (Model 14), we tested whether the mediation effect of executive function on the relationship between gross motor skills and emotional regulation is moderated by psychological resilience. The results confirmed Hypothesis 3. As shown in Table 5, after the inclusion of psychological resilience in the model, the interaction effect between executive function and psychological resilience was significant (β = 0.078, t = 2.164, p < 0.05). This finding indicates that psychological resilience moderates the latter part of the mediation effect, suggesting that the indirect impact of gross motor skills on emotional regulation through executive function is influenced by psychological resilience during the final stage of the pathway.

**Fig. 2:**
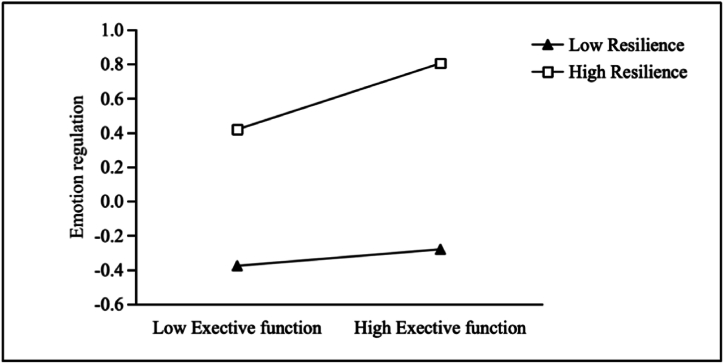
Moderating effect of resilience on the relationship between executive function and emotion regulation.

Psychological resilience was categorized into three groups (high, medium, low) based on one standard deviation above and below the mean. A simple slope analysis was conducted to interpret the moderating effect. The results (Fig. 2) indicated that for individuals with lower psychological resilience (M − 1 SD), the positive predictive effect of executive function on emotional regulation was not significant (β = 0.051, t = 0.931, p > 0.05). In contrast, for individuals with higher psychological resilience (M + 1 SD), the positive predictive effect of executive function on emotional regulation was significant (β = 0.202, t = 3.828, p < 0.01). These results show that the increase in psychological resilience leads to a shift in the direct impact of executive function on emotional regulation from being nonsignificant to significant and becoming progressively stronger.

The indirect effects of executive function on emotional regulation were examined under the moderating effect of psychological resilience. Bootstrap sampling (Sample = 5,000) was performed for psychological resilience levels one standard deviation above and below the mean, as shown in Table 6. The results indicated that when psychological resilience was high (M + 1 SD), the mediation effect of gross motor skills on emotional regulation through executive function was significant. By contrast, when psychological resilience was low (M − 1SD), this mediation effect was not significant. These findings suggest that when psychological resilience is high, executive function and emotional regulation are strongly positively correlated. Thus, this study concludes that psychological resilience enhances the impact of executive function on emotional regulation, indicating that psychological resilience and executive function jointly affect emotional regulation.

**Table 6:** The mediating effect of executive function between fundamental motor skills and emotion regulation at different levels of psychological resilience.

	Resilience	Effect	BootSE	BootLLCI	BootULCI
	eff1(M-1SD)	0.051	0.055	−0.056	0.158
The moderated mediation effect	eff2(M)	0.127	0.041	0.047	0.207
	eff3(M+1SD)	0.202	0.053	0.098	0.306

## 5 Discussion

The findings of the present study support the hypotheses, indicating that gross motor skills positively affect emotional regulation, with executive function serving as a mediator in this relationship. Furthermore, psychological resilience acts as a moderator, strengthening the effect of executive function on emotional regulation and thereby enhancing emotional management.

### 5.1 Relationship between gross motor skills and emotional regulation

Gross motor skills were significantly associated with emotional regulation skills among Chinese rural preschoolers. This finding is consistent with those of previous studies [[71], [72], [73]] that have reported a strong relationship between motor skills and the development of socioemotional skills in children with developmental difficulties and disorders. A possible explanation for this finding is that children with higher motor skills tend to be more physically active, which in turn supports their emotional regulation. Enhanced postural control allows them to explore and interact more effectively with their environment, improved locomotor skills enable them to access new spaces, and better object control skills provided them with more opportunities for engaging with different objects and participating in social interactions [74]. Hyde suggested that regular physical activity can enhance positive emotions across different age groups, including in children (aged 3–9 years), adolescents (aged 10–17 years), young adults (aged 18–25 years), adults (aged 26–65 years), and seniors (aged 65 years and above) [75]. Other studies have also demonstrated that children who engage in physical activities have stronger emotional regulation capabilities compared with their peers who do not participate in such activities [76]. This may be explained by the role of physical activity in stress management and emotional regulation. Moderate-intensity physical activities can trigger the release of endorphins, which promote a pleasant mental state and reduce negative emotions, such as depression, anxiety, and tension. Furthermore, physical activities help children build confidence and a sense of self-efficacy, which are crucial for better emotional regulation. Children who are more confident in their abilities are better equipped to handle challenges and regulate their emotions in difficult situations [77].

Research on the relationship between gross motor skills and socioemotional behavior in typically developing children is limited and has contradictory results [[78], [79], [80]]. Salaj and Masnjak found a significant but weak correlation between motor skills and socioemotional functioning in typically developing preschool children [81]. However, the generalizability of their findings is limited by their small sample size of 125 children from public kindergartens. In addition, most of the previous research has focused on special populations, such as children with autism spectrum disorders or developmental coordination disorder, resulting in less conclusive evidence for typically developing preschool children [82]. Moreover, the use of diverse assessment tools that emphasize different aspects of motor skills and socioemotional skills has contributed to inconsistencies in research outcomes [83,84].

In summary, developing gross motor skills through physical activities can positively influence children's emotional development, especially their ability to regulate emotions. However, further research is needed to better understand the relationship between motor skills and socioemotional functioning in typically developing preschool children.

### 5.2 Mediating role of executive function in fundamental motor skills and emotional regulation

Understanding how fundamental motor skills affect emotional regulation necessitates examining the mechanisms underlying the relationship between these two factors. Specifically, identifying mediating variables can clarify the effect of motor skills on emotional regulation. This study investigated the mediating effect of executive function on the relationship between fundamental motor skills and emotional regulation among rural preschool children. The results indicated that executive function positively predicts the emotional regulation ability of rural preschool children; this result is consistent with the findings of previous studies [37,38]. Executive function acts as a “bridge” in the relationship between motor skills and emotional regulation in rural preschool children [85]. It affects emotional regulation directly, as well as indirectly by modulating executive function.

Drawing on Piaget's cognitive development stage theory [86] and dynamic systems theory [87], we can better understand the role of executive function in the relationship between fundamental motor skills and emotional regulation. According to these theories, early motor development in children is a crucial indicator of their intellectual development (executive function) and substantially affects their psychological development (emotional regulation). Motor skills enable children to actively construct and engage in their own growth [88,89]. These theories provide theoretical support for exploring how motor skills influence executive function and, subsequently, emotional regulation.

Neuroscience research indicates that short-term aerobic exercise can elevate neuroendocrine levels in the brain, leading to changes in the levels of neurotransmitters, such as dopamine, norepinephrine, and epinephrine. These neurochemicals play a significant role in influencing emotions and are crucial for emotional regulation. In addition, exercise improves wakefulness, increases blood flow to the prefrontal cortex, and enhances executive function. Exercise also affects brain structures linked to emotional states, such as the anterior cingulate cortex, hippocampus, dorsolateral prefrontal cortex, and amygdala. These changes help individuals manage adverse emotions and experience greater emotional stability and pleasure [90,91]. The overlap in brain mechanisms activated by exercise—affecting both executive function and emotional regulation—suggests that improving executive function through physical activity positively influences emotional states. Therefore, for rural preschool children facing challenging circumstances, interventions aimed at promoting both fundamental motor skills and executive function may effectively enhance emotional regulation.

### 5.3 Moderating role of psychological resilience in executive function and emotional regulation

The present study discovered that psychological resilience moderates the latter part of the mediation effect of executive function in the relationship between fundamental motor skills and emotional regulation. Psychological resilience, which refers to an individual's ability to adapt and recover from setbacks and stress, is crucial for overall development. The results showed that children with higher levels of psychological resilience experience a stronger positive effect in the mediation process where executive function links motor skills to emotional regulation. By contrast, correlations in this mediation pathway are weaker in children with low levels of psychological resilience.

### 5.4 Limitations

Our study has several limitations. First, the sample was predominantly from rural areas in Southern China, which may limit the generalizability of the findings to other regions or cultural contexts. Second, the cross-sectional design of the study makes it difficult to establish causal relationships between gross motor skills and emotional regulation. To address this, future research should include longitudinal and experimental studies to explore these causal links more thoroughly. Additionally, other variables not examined in our study could significantly influence the observed relationships. For example, factors such as the family's socioeconomic status and parent educational levels were not considered and may contribute to potential model misspecifications.

## 6 Implications and future research

This study has significant implications for early childhood education and the promotion of young children's health. In general, a strong emphasis is placed on students' academic achievement, which can overshadow the importance of physical activity and exploration. However, physical well-being cannot be fully achieved without dedicating time for both outdoor and indoor physical activities. The study highlights the importance of engaging young children in various physical activities, such as running, sliding, climbing, jumping, and playing with balls, because these activities are crucial for their overall physical development. Thus, parents should prioritize their children's physical well-being by encouraging them to engage in a diverse range of sports and physical activities.

In terms of early childhood education practice, the current state of environments and curricula designed to promote children's physical well-being in rural China is inadequate. Research indicates that outdoor settings in many early childhood education facilities are subpar, largely due to a lack of stationary and portable equipment that can foster the development of various motor skills. Additionally, large class sizes in most rural preschools exacerbate the problem because such settings entail children to take turns due to limited outdoor equipment, such as swings, slides, and balls. This results in reduced opportunities for vigorous physical activity and limits the overall effectiveness of these programs in promoting physical development.

Future research should investigate the differences and similarities in fundamental motor skills, executive function, psychological resilience, and emotional regulation across various age groups and cultural backgrounds. Moreover, conducting intervention studies could validate the impact of fundamental motor skills on children's emotional regulation and provide deeper insights into the mediating and moderating roles of executive function and psychological resilience.

### 6.1 Recommendations

The findings from this study can guide the creation of targeted interventions and policies to enhance the health and holistic development of preschoolers in rural China, addressing disparities in the physical, cognitive, and socioemotional outcomes. To improve both physical and emotional well-being, a collaborative approach involving policymakers, educators, and parents is essential. Policymakers should prioritize investments in infrastructure and resources that support the physical and mental health of preschools, especially in underserved rural areas. This includes developing safe, engaging environments with facilities that promote motor skill development and allocating resources for mental health support services. Educators need specialized training programs [92] to integrate physical games and activities into daily routines effectively, fostering emotional regulation and overall well-being. Emphasizing gross motor skills in the curriculum will support children's holistic development, contributing to both their physical and emotional health. Parents play a crucial role in balancing academic preparation with physical activities at home. Encouraging outdoor play and games can significantly contribute to their children's comprehensive growth [93]. Active parental involvement helps extend the benefits of physical and emotional development beyond the classroom, ensuring ongoing support for children's well-being.

## 7 Conclusion

The findings of this study highlight the crucial role of psychological resilience in mediating the relationship between fundamental motor skills and emotional regulation, specifically through executive function. Children with higher psychological resilience are better able to handle setbacks and adapt to changes, which enhances their ability to use executive function for effective emotional regulation. By contrast, children with lower psychological resilience may have less effective emotional regulation due to weaker executive function support. These results emphasize the need for educators to focus not only on developing executive function but also on fostering psychological resilience. By adopting a comprehensive approach that addresses both of these aspects, educators can more effectively enhance children's emotional regulation abilities.

## Data availability statement

Data are available from the authors upon reasonable request.

## Funding

This work was supported by National Institutes of Education Science (Project Number: BHA190149), and the Specialized Project on Social Service Research of Guangdong Teachers College of Foreign Languages and Arts (Project Number: 2022ZX27)

## CRediT authorship contribution statement

**Xinyue Ma:** Writing – review & editing, Writing – original draft, Visualization, Formal analysis, Data curation, Conceptualization. **Ning Yang:** Writing – review & editing, Project administration, Conceptualization. **Meixian Huang:** Software, Resources, Methodology. **Shuwei Zhan:** Investigation, Formal analysis. **Houwen Cao:** Writing – review & editing, Methodology, Formal analysis. **Shan Jiang:** Writing – review & editing, Supervision, Methodology, Formal analysis, Data curation, Conceptualization.

## Declaration of competing interest

The authors declare that they have no known competing financial interests or personal relationships that could have appeared to influence the work reported in this paper.
